# Explaining the experiences of nursing administrators, educators, and students about education process in the COVID-19 pandemic: a qualitative study

**DOI:** 10.1186/s12912-021-00666-4

**Published:** 2021-08-20

**Authors:** Zahra Farsi, Seyedeh Azam Sajadi, Effat Afaghi, Andrew Fournier, Shahla Aliyari, Yazdan Ahmadi, Ebrahim Hazrati

**Affiliations:** 1 Medical-Surgical Nursing, Research and Community Health Departments, Faculty of Nursing, Aja University of Medical Sciences, Tehran, Iran; 2 Department of Nursing Management, Faculty of Nursing, Aja University of Medical Sciences, Tehran, Iran; 3 Department of Medical-Surgical Nursing, Faculty of Nursing, Aja University of Medical Sciences, Tehran, Iran; 4 College of Doctoral Studies, Grand Canyon University, Phoenix, AZ USA; 5 Department of Maternal Newborn Health, Faculty of Nursing, Aja University of Medical Sciences, Tehran, Iran; 6 Department of Emergency Nursing, Faculty of Nursing, Aja University of Medical Sciences, Tehran, Iran; 7 Critical Care Medicine, Trauma Research Center, Aja University of Medical Sciences, Tehran, Iran

**Keywords:** Pandemic, COVID-19, Education, Nursing, Qualitative Study

## Abstract

**Background:**

The coronavirus disease 2019 (COVID-19) has severely influenced various aspects of human life, particularly education. This study aimed to explain the impact of the COVID-19 pandemic on nursing education from administrators, educators, and students’ perspectives.

**Methods:**

This qualitative study with a conventional content analysis approach was conducted from June to October 2020 at a nursing school in Tehran. Thirteen participants were enrolled using purposive sampling. Data collection was through in-depth and semi-structured interviews and continued until reaching data saturation. Nursing administrators, educators, and students constructed interviews to understand nursing education changes during the pandemic. All interviews were recorded, transcribed, reviewed, coded, and analyzed using the Graneheim and Lundman methods.

**Results:**

Interviewed respondents included administrators and professors (*n* = 6) and nursing students (*n* = 7). The respondents reported five main topic areas: (1) safe management in ambiguous situations; (2) perceived situations; (3) adaptive coping; (4) educational facilitators and challenges, and (5) continuing education in an uncertain context. The central theme was “close conflict of education with COVID-19”.

**Conclusions:**

The current study noted instability and challenges placed on nursing education during the pandemic. Opportunities were addressed during the pandemic to improve the nursing training process using planning, scientific management, emerging technology, innovative educational opportunities, and comprehensive support from institutional stakeholders. Clear guidelines and recommendations are needed to ensure medical education safety during the pandemic.

## Background

Novel coronavirus (nCoV), is also called coronavirus disease 2019 (COVID-19). The first reported outbreaks occurred in China in December 2019. The World Health Organization declared COVID-19 as a pandemic on March 11, 2020 [1]. As of July 6, 2021, there were 183,700,343 infected individuals and 3,981,756 cases of death reported due to COVID-19 worldwide [2].

Two confirmed cases were reported in Qom, Iran, on February 19, 2020 [1] and were imposed suppression and lockdown in all the provinces after confirming new cases [3]. Iran observed an increase in the number of cases from the beginning of May, signaling a second wave [4], and entered its third wave in October 2020 [5].

In just a few minutes, the nCoV can be spread from infected cases to others through droplets resulting from upper respiratory secretions and touching contaminated surfaces and objects. Thus, all individuals are susceptible to this virus’s long-term side effects [6]. The COVID-19 pandemic has also led to numerous education challenges [7]. In this sense, educational centers, including higher education institutions, are at an increased risk of the nCoV spread due to overcrowded classrooms and protective measures’ complexity [8]. From early April 2020, many students in 194 countries across the world have stayed home because of the closures of educational centers. This pandemic has had significant impacts on students’ life and work and their social life, financial status, and emotional health [9]. Higher education institutions have been gradually influenced by direct rules and regulations, including classroom lectures cancelations, restrictions on working activities, physical distancing and teleworking, and distance education approaches [8]. At the beginning of this pandemic, many businesses and organizations were locked down, and higher education institutions were no exception. However, this condition allowed all countries to change their education system and concentrate on emerging technologies. Strengthening education programs and taking health measures in higher education institutions can help students and other beneficiaries prevent the nCoV from spreading and maintaining the education process [6]. In times of this pandemic, the universities of medical sciences have also been facing more severe challenges since they train the next generation of healthcare providers [10]. In this respect, nursing and medical students are among groups forced to deal with global health issues [11]. Continuation of education in medical universities in the COVID-19 pandemic to train professional and empowered graduates to reduce the workload of hospitals is one of the main concerns of educational administrators [12].

Interruptions to theoretical and clinical education following the COVID-19 pandemic were unexpected for nursing students. Evidence suggests that no awareness of time, place, and manner of education to compensate for deficiencies is overloading these students with too much stress [13]. Once faced with a pandemic, nursing students experience excessive mental pressure and even feel worried about their future jobs. They live through the feelings of thrill, uncertainty, and helplessness [14]. Despite the educational challenges, this exceptional situation provides a good opportunity for nursing students to learn, take initiative, and be creative [15].

Although nursing students may take theoretical and general courses in the academic environments, they need to spend much time in a clinical internship [16]. Nursing administrators and educators must meet the educational needs of such students. For this purpose, it is essential to recognize these students’ experiences and expectations in the face of such changes to contribute to education, resource allocation, and re-orientation of academic instruction [17]. The results of an investigating of the clinical education experiences of Belgian nursing students during the COVID-19 pandemic period showed that some students described it as an enriching and constructive educational situation and others as an unsafe, stressful, difficult, and overwhelming situation [15].

COVID-19 is a crisis giving real lessons of fairness, leadership, social judgment, ethics, and patient care to humans. This pandemic has also affected future visions of education [10]. Experts believe that leaders in times of crisis can assume two essential responsibilities, i.e., to solve problems immediately and prevent their recurrence, wherein the former has been overemphasized, and the latter can have long-term consequences [14]. Therefore, leaders and those in charge need to pay special attention to decisions made during crises. In this respect, in a mission-centered nursing school in Iran, there were attempts to prevent delay and not interrupt the education process, unlike other schools and educational centers. Regarding its missions and specific educational policies and security issues and discretion by upstream administrators, while examining existing possibilities, conditions, and infrastructure, and observing health protocols, this school benefitted from a combined (namely in-person and virtual) education program. A recent study reported that over 66 % of students in the mentioned school were satisfied with the quality of the training course and more than half of the students (56.3 %) were relatively satisfied with virtual education [12]. Regarding the current conditions, the research question addressed was: “*What are the experiences of nursing administrators, educators, and students about the COVID-19 pandemic*?” The review of the related literature revealed no study, to the best of authors’ knowledge, reflecting on this subject-matter in education centers during this crisis. Considering the importance and lack of studies, the researchers attempted to make use of content analysis to boost the education process for students in this mission-centered nursing school in times of the COVID-19 pandemic, whose results were reported in the present study to explain the impact of the COVID-19 pandemic on nursing education from administrators, educators, and students’ perspectives.

## Methods

### Design and setting

This qualitative study with a conventional content analysis approach was conducted from June to October 2020 at a nursing school in Tehran, Iran.

### Participants and sampling

Thirteen participants experiencing the phenomenon under study, including nursing administrators, educators, and students recruited based on the purposive sampling technique. The inclusion criteria were rich experience under the study objectives, fluency in speaking, and willingness to participate in the study and share experiences. The exclusion criteria also comprised of unwillingness to cooperate and continue the study. No one of the participants refused or withdrawn after giving their agreement. The researchers looked for those with a rich experience of the phenomena under the study and have the ability and desire to express it. The first participant was a faculty member with education and management experience in the COVID-19 pandemic.

### Data collection and analysis

In this study, data collection and analysis were performed simultaneously during the study period. The interviews were conducted in quiet places such as administrators’ and educators’ offices and at the Vice-Chancellors’ Office for Research in the school. The data were collected using semi-structured interviews with open-ended questions, for example: “*How do you evaluate* the *education process in the last semester?”, “What are the differences between current conditions and the ones before the COVID-19 pandemic?”, “What strategies do to take to maintain* the *education process?” “Have you ever faced challenges in* the *education process?”, “What have you so far done to deal with challenges to* the *education process?”* The main questions were designed by the researchers. The participants were asked to provide more examples or to clarify their reasons for describing the topic in question. Probing questions were raised to understand the detailed experiences. For instance, “*How do you think these challenges occur?*” and “*What do you mean by that?*” After obtaining informed oral consent from the participants, the study’s primary objectives were repeated at the beginning of each interview, and the interviews were recorded. The use of the interview method for data collection is one of the most appropriate ways to avoid recall bias. The first and corresponding authors conducted the interviews. Each interview’s duration depended on the information and the participants’ willingness and agreement to share their experiences. The interviews’ total time was 395 min with a mean of 36.87 min (ranged from 26 to 53 min). The interviews also continued to reach data saturation, meaning no further data and new concepts were obtained on the topics of interest. The field notes also were used as another data collection strategy.

**Fig. 1:**
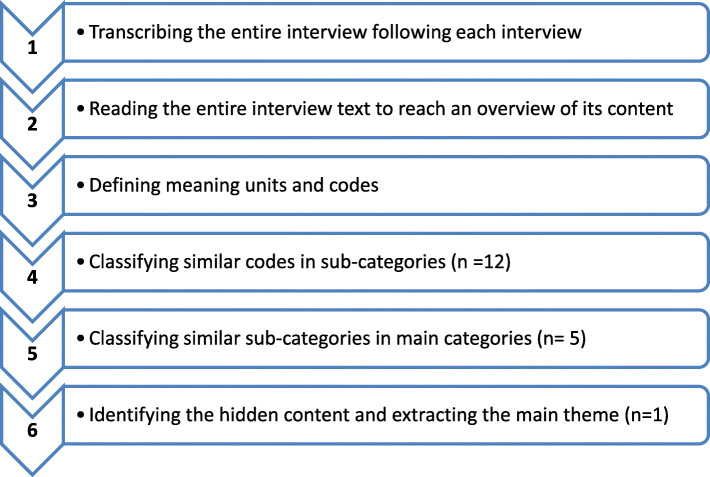
Flow diagram of data analysis steps

In this study, an inductive approach was applied. All the recorded interviews were transcribed verbatim in Microsoft Word 2017, and data content analysis was performed based on the steps proposed in the Graneheim and Lundman method by two researchers (first and corresponding authors with experience in qualitative data processing). Ambiguities were resolved by checking the transcripts with the participants immediately after the interview. Under the Graneheim and Lundman method for qualitative data analysis, the transcripts and field notes were repeatedly read several times, reduced to units of meaning, and then labeled with initial codes. The extracted codes were compared, referring to their similarities and differences, and divided into sub-categories and categories. This trend continued across all the analysis stages until theme emerged (Fig. 1).

The researchers used manifest and latent content analysis. Constant comparison analysis as one of the principal qualitative content analysis approaches was utilized to review the extracted sub-categories and categories. Memoing, as another technique, was further employed during the content analysis. The researchers independently and inductively created codes, sub-categories, and main categories.

### Trustworthiness

Guba and Lincoln’s criteria, which included credibility, transferability, dependability, and confirmability, were considered to increase the study’s trustworthiness [18, 19]. To enhance the credibility, the researchers considered diversity in sampling, established relationships with the participants, used data collection and researcher triangulation, persistent observation, searched for documents and evidence, analyzed negative cases, reflected on the credibility of the researchers (all the researchers were engaged in education or managerial affairs in the study setting during the COVID-19 pandemic). Besides allocating enough time to data collection and analysis and to immerse in the data were used to validate the data. Also, the findings were approved by some participants (member check) and two qualitative method researchers (peer check). To increase transferability, a complete description of the entire study process and context was provided and participants were quoted directly. To increase dependability and confirmability after completing individual data analyses, all of the researchers tried to jointly achieve a definitive organization of data, so a common consensus was ultimately reached. Also, all the study steps were recorded and a report of the study process was prepared.

### Ethical consideration

 The present study received approval from the Ethics Committee of the Aja University of Medical Sciences, Tehran, Iran, with the code: IR.AJAUMS.REC.1399.066 on June 6, 2020. The Declaration of Helsinki was considered in this study. For instance, written and oral consent was also obtained from all participants to participate in the research and recording of their interviews.

Participation in the study was voluntary, and there was no pressure or encouragement for their inclusion. The participants were also ensured about the data’s confidentiality and the right to withdraw from the study. Also, the Committee on Publication Ethics was considered.

## Results

Out of thirteen participants recruited in this study, nine cases were female. The mean age of them was 36.07 years. In terms of education levels, an educator was holding Ph.D. degrees, two educators were Ph.D. degrees, and three had master’s degrees. There were also four master’s degrees and three bachelor’s degree students, respectively. Six participants were faculty members with education and management experience at various levels, from department heads to nursing school dean, and seven participants were students (Table 1). The educators’ age range was 39–56 years old with 15–29 years of teaching experience. The students were also aged 21 and 38 years old. The study findings included 785 initial codes, 12 sub-categories, and five categories under one theme, as depicted in Table 2.

**Table 1:** Characteristics of the Participants

Participant	Sex	Education level	Educator	Administrator	Student
P1	Female	Ph.D.	*	*	-
P2	Male	Ph.D.	*	*	-
P3	Female	MSc	*	*	-
P4	Female	MSc	*	*	-
P5	Female	MSc	*	*	-
P6	Female	Ph.D. Candidate	*	*	*
P7	Female	BSc student	-	-	*
P8	Male	MSc student	-	-	*
P9	Male	MSc student	-	-	*
P10	Female	MSc student	-	-	*
P11	Male	MSc student	-	-	*
P12	Female	BSc student	-	-	*
P13	Female	BSc student	-	-	*

**Table 2:** Main categories and sub-categories

Main theme	Main Categories	Sub-Categories
Close conflict of education with Covid-19	Safe management in ambiguous situations	Provision of health safety grounds
		Requirements for successful confrontation
		Outcomes
	Perceived situations	Underlying conditions
		Perceived stress
	Adaptive coping	Experience and coping with situations
		Collaboration
	Educational facilitators and challenges	Educational facilitators
		Educational challenges
	Continuing education in an uncertain context	Gradual formation of education in a virtual context
		Unstable clinical education
		Unstable theoretical education

### A. Safe management in ambiguous situation

The first category obtained from the data in this study was “safe management in ambiguous situations,” comprised of the sub-categories of “provision of health safety grounds,” “requirements for successful confrontation,” and “outcomes.”

#### Provision of health safety grounds

During the study, no vaccines and certain medicines were introduced for COVID-19. The most important way to control this condition was to respect health protocols. The study participants pointed out cases such as screening, isolation of students with confirmed infection, distribution of disinfectants, restrictions on students’ travels, social distancing in dormitories and classrooms, deployment of a physician in the nursing school, changing internship uniforms and shoes in separate dressing rooms, training and obliging students on how to observe health protocols, allocation of face masks to students throughout internship programs, and control of students’ daily body temperature in the school of nursing. In this respect, one administrator stated:

“*We firstly did the screening and took PCR tests from all students through coordinating with the Vice-Chancellor’s Office for Health. We also marked the chairs in the classrooms alternately to respect social distancing. All classrooms were additionally disinfected by janitorial servicemen every day. Face masks and disinfectants were also distributed among the students, and the isolation room was then prepared. The general practitioner, fully equipped, was ultimately deployed in the physical education room for five days, and all students were examined.*” (P_1_).

Another participant said:

“*The students were wearing face masks, even during their courses, and were using alcohol for disinfection purposes. The administrators were repeatedly referring to the classrooms during rest times to give notices and the number of individuals in the classrooms also reduced by half.*” (P_8_).

#### Requirements for successful confrontation

Medical sciences graduates are involved in human life, so community health depends on the quality of education provided by the medical sciences universities. Regarding this vital mission and the necessity of maintaining educational programs, restrictions arising from COVID-19 must be an opportunity to exploit existing capacities. To reach the desired status, the study participants mentioned cases such as management of overcrowded classrooms, clinical courses, and dormitories through proper planning, allocation of particular hospitals to patients suffering from COVID-19 to boost the capacity of clinical education, use of combined (namely, in-person and virtual) education, utilization of alternatives such as workshops and simulation, increased Internet bandwidth, provision of healthcare equipment, applied research development, presence of a psychologist in the nursing school, virtual library system development, managerial requirements like transparency and courageousness in deciding by administrators, unity of command, consideration of criticism raised by staff, justification of opinions by staff and students and their involvement in decision-making, emphasis on encouraging educators and staff, virtual content monitoring, adherence to the law instead of taste-orientedness, mutual understanding, and promotion of communication skills. One participant said:

“*I think we are now in a time of crisis, and there is a need to live and work during this pandemic. It is of no use to interrupt classrooms, internship programs, and even education process merely because of such risks.*” (P_2_).

The same participant also reiterated:

“*It is better if we interact with the staff. We also need to be transparent and involve them to assume responsibilities and to learn about the problems. If we do not share challenges with the staff and have no interactions, numerous problems will arise.*” (P_2_).

#### Outcomes

Active attendance and emphasis on the nursing school’s continuous education process could lead to excellent outcomes even in the face of difficulties. The study participants referred to cases such as minimal interruption in clinical education, continued theoretical education, combined education, removal of educational deficiencies, improvement of health conditions in dormitories, the tendency of some schools to imitate the nursing school examined, higher levels of awareness, and respect for health and consideration of social distancing within the nursing school campus. For example, one of the study participants stated:

“*We thank God that field students could get along, and even no one objected about taking internship programs. In the meeting held by the Vice-Chancellors’ Office for Education with the presence of authorities from other universities, they had stated that they were following our policies because they believed that their students had been lingering clinically, and they could not be graduated in time.*” (P_1_).

In this line, one asserted:

“*The field students completed their internship programs with too much fear and anxiety. Once personal protective equipment such as face masks and gloves were distributed among the students, they could feel more relaxed.*” (P_5_).

One participant similarly added:

“*In the present situation, everything has become much better. There is no fear and anxiety-like that at the beginning of the pandemic, and the planning is more coherent.*” (P_3_).

### B. Perceived situation

The second main category extracted from this study was “perceived situation,” consisting of the sub-categories of “underlying conditions” and “perceived stress.”

#### Underlying conditions

Understanding is a prerequisite to fulfill tasks and making correct decisions. Since the SN examined in this study was organization-based and mission-centered with essential objectives such as training responsive experts to deal with crises, there were attempts to respect health protocols and maintain clinical education even though virtual education also benefitted. In this organization, the hierarchical structure had particular importance, and deference to upstream directives was an obligation. Considering the underlying conditions in the nursing school, the participants reflected on cases such as courses for organizational learning beyond those approved by the Ministry of Health and Medical Education (MoHME), recruitment of organizational staff from the nursing school graduates, constant attention to promoting capabilities in educators, the priority of in-person education and traditional methods of teaching, continuous evaluation of educators’ performance, educational stability before COVID-19 prevalence, adherence to instructions from high-ranking officials, emphasis on good order and discipline by students, and supervision over the maximum presence of students in educational programs. In this vein, one said that:

“*Prior to the pandemic, the condition was much more stable. We were teaching based on the program from the beginning of the semester to its end. There were no big changes but slight ones. For example, the time and the hours of the classrooms could be changed for one session, but now conditions have become different*.” (P_4_).

One administrator also noted:

“*Since the essence of our university is military, students know that this educational center is different, and they have to observe all rules and regulations. They should even know that they are military nurses, and they need to be on the frontline in times of wars and crises. They were justified and convinced but staying in the dormitory and not leaving there even for small purchases was annoying.*” (P_1_).

Another administrator similarly stated:

“*We are a special university, and the existing rules and regulations within this organization are different from national ones. Sometimes some guidelines are different from university-level ones. For example, other universities were locked down from February 20, 2019, but the students enrolled in our university had to attend classrooms and complete the courses until March 7.*” (P_2_).

#### Perceived stress

Being drawn against an unknown and health-threatening situation can cause stress. As a presence in social events could lead to increased risk of infection, the need for maintaining the education process in-person and clinically and residing in dormitories might impose too much stress and multiply the incidence rates of specific reactions and behaviors among educators and students, particularly at the beginning of the pandemic. The participants highlighted cases such as an increase in complaints, worries, and fears among educators and students regarding being infected with COVID-19, tensions of living in dormitories due to fear of catching the virus, senses of uncertainty, confusion, and panic, and worries among educators to transfer the disease to their children and family through their commutes to the nursing school and hospital, concerns about an ambiguous future, mental pressures following numerous organizational instructions, students’ concerns about the decline in education quality, resistance by some educators and students against attendance in classrooms and internship programs, mental pressures following quarantine, and diminished motivation. One of the postgraduate students said:

“*I think some teachers and postgraduate students here had a phobia. The students were working in the hospital-specific to patients suffering from COVID-19. This led holding (to holding) virtual classrooms and extended to other courses and programs.*” (P_7_).

One of the postgraduate educators reiterated:

“*Firstly, there was a state of confusion and chaos. We were all in a rush and felt stressed out. We even had anxiety. We did not know about the disease. We even did not know about this unknown pandemic and its influx to Iran. As we were working in the hospital, we could get informed about figures and statistics for infected cases in China and subsequent quarantine. There was a kind of horror. There was also fear, and one of the strongest feelings at that time was anxiety due to this event. I was afraid of being infected and spreading the virus to my children. I was really concerned about my family*.” (P_3_).

### C. Adaptive coping

The third category extracted from the data in this study was “adaptive coping,” encompassing the sub-categories of “experience and coping with situations” and “collaboration.”

#### Experience and coping with situations

Some participants emphasize cases such as changes in attitudes towards the importance of and respect for health protocols, the supply of personal protective equipment, gradual stress removal, more dominance and control over existing conditions, attempts to cope with crises, efforts to turn threats into opportunities, lower resistance against changes, finding strategies for favorable compatibility, adaptability to conditions by authorities, acceptance of situations, gradual progress in educational programs following gaining experience, and experience acquisition after encounters. One educator said:

“*Problems make us find a way and use it as an experience in the future. We need to look at this issue optimistically. We should not see this condition as a disease-causing many problems. If some events, good or bad, happen in the future, we can make use of our experience*.” (P_5_).

She added:

“*Management of a problem that suddenly arises is really valuable. Being a good teacher does not mean everything is available, but it is of utmost importance to be a good teacher with shortages. I think this is of value*.” (P_5_).

#### Collaboration

One requirement to cope with crises, including pandemics, is the strength of mind working in partnership to achieve objectives. Taking advantage of this potential can direct a school. In this respect, the participants mentioned cases such as collaboration by field students to monitor health status in dormitories, division of work among students to evaluate symptoms of suspected cases, cooperation between authorities and Vice-Chancellor’s Offices in the nursing school, the responsibility of senior students to support those undergoing quarantine. The participants also spoke of staff teamwork, efforts by educators to provide proper education, programs for educators to attend hospitals to help in clinical nursing, contributions to students under quarantine based on health protocols, the active presence of educators in in-person and virtual education, the participation of educators to maintain and promote students’ spirit, and responsiveness to students’ concerns by the education department staff via phone calls or in person. An undergraduate student said:

“*It was decided to manage the dormitory at first because we were among senior students. In fact, we managed the dormitory as we were in evening and night shifts. We were divided into groups to check the students’ body temperature every night. We were also supervising the kitchen and the bathrooms and cleaned them with specific bleaches. We additionally controlled hand wash by the students before and after classrooms and internship programs. They were required to throw their used masks inside trash bins. Those in charge used to clean the surfaces, chairs, and doorknobs with water and bleaches because disinfectants were in shortage.*” (P_6_).

### D. Educational facilitators and challenges

The fourth category obtained in this study included “educational facilitators and challenges” comprised of “educational facilitators” and “educational challenges.”

#### Educational facilitators

During the COVID-19 pandemic, several factors facilitating the education process are maintained in the SN. The participants shed light on cases such as requirements for adherence to organizational rules and regulations by the students, the possibility to establish a direct relationship between students and school head, hierarchical structure, efforts by educators to encourage students in in-person classrooms, the usefulness of instructions for biological defense, attracting sponsors to provide healthcare equipment, management support, coordination between educators and education department heads, obedience to commanders by students and educators, tendency to upgrade the quality of education, sense of responsibility for students’ health by educators, a transition of educators’ experiences to students, availability of educators, and participation of educational advisors to direct students towards academic goals. In this respect, one of the undergraduate students reiterated that:

“*In my opinion, our teachers help us a lot. The information, experiences, and pieces of advice, as well as the latest scientific content provided by our teachers, were as facilitators. We trusted them, and they helped us*.” (P_6_).

In this line, one educator noted:

“*I think management support is of utmost importance. We need to have unity of command in decision-making, and we are also in need of support. If decisions are made, they can be implemented, provided that there are upstream orders. As a whole, the support by managers and commanders always matters*.” (P_2_).

He added:

“*Educators’ compliance with the decisions made at the school level is contributing. For example, we could not complete this semester if they had not given a hand*.” (P_2_).

#### Educational challenges

Despite facilities, there were numerous challenges in maintaining the education process, decelerating movements towards educational goals. In this sense, the participants referred to cases including no respect for health protocols by some students, non-perception of risks by some students, limitations in terms of adherence to physical distancing in dormitories and classrooms, delays in fulfillment of theses and research projects, problems facing postgraduate students in sampling for their theses, increased work pressures on in-service postgraduate students, lack of experienced counselors, psychologists, or psychiatrists in the school, limited provision of protective personal equipment for students, doctoral students’ employment in hospitals, no stability in decision-making by some administrators due to unstable conditions, resistance by some students and educators against continuous education, wrong beliefs about health among some students, multiplicity of commands, improper ventilation in some classrooms, hasty decisions, unavailability of infrastructure to provide virtual contents, no access to information technology experts, high costs of the Internet, and expensive personal protective equipment. One administrator said:

“*The main challenge bigger than the ones I experienced is that orders and decisions are made in haste. Now, during these hours, they tell me to do something, and I do it, but they tell me to cancel it and do something else*.” (P_2_).

And another stated:

“*One of the major problems is how to provide personal protective equipment. They are really expensive, and hospitals cannot afford to give them to students. Our university is no exception. So, we cannot send the students to the wards for patients with COVID-19. We have to deploy them in general wards only with a pair of gloves and a face mask. Accordingly, the diversity and the number of patients are lower theoretically and clinically. Education quality is thus low*.” (P_1_).

One educator declared:

“*It was kind of a tough experience. We did not have the facilities; for example, a recording was a real problem, and we had no place to do it. We had to provide recorded files at home, and we needed to tell others around to be quiet. It was also real stress to create the slides. The main problem was that we had no format to make the slides. For example, adding voice to the slides could make the size of the files so large that they could not be attached in an e-mail. Then, they provided us with specific formats for such slides, and their size and everything became better. One more thing was that we could not communicate with the students*.” (P_3_).

### E. Continuing education in an uncertain context

The final category extracted in this study was “continuing education in an uncertain context,” containing the sub-categories of “gradual formation of education in a virtual context,” “unstable clinical education,” and “unstable theoretical education.”

#### Gradual formation of education in a virtual context

The COVID-19 pandemic provided educational centers with an opportunity to use new teaching methods and then exploit new technologies. There were also attempts in this nursing school to turn existing threats into virtual education opportunities even though there were problems. The participants focused on cases in which students encountered technical issues with access to virtual education files, lower quality of virtual education compared to in-person sessions, low quality of virtual files due to a lack of skill by some educators at the start of the pandemic, no opportunities for questions and answers during virtual sessions, lack of infrastructure for virtual education, lack of satisfaction in adding vocal support for PowerPoint slides, organizational limitations during online sessions, inadequate facilities to support the addition of voices to the slides, difficulty in the transition of students to virtual education, delivery of a deluge of audio slides to students in a short time, and student fatigue due to feelings of being overworked in virtual and in-person classrooms. The problems also created opportunities to enhance virtual education, which included efforts by department heads to control virtual files, feedback to educators by the Vice-Chancellor’s Office for Education about the quality of the slides and emphasis on their correction, the establishment of a new system called NAVID, uploading files for students, notifying students to use this NAVID, taking online tests and delivering numerous assignments to students, responses to student issues and problems, encouraging students to use the system, and removal of organizational limitations regarding online theoretical courses. A participant said:

“*During in-person education, educators and students are in a physical space, then educators teach, and the exchange of opinions and transparency consequently occur. Accordingly, ambiguities are met. But the questions are merely raised offline if the courses are virtual, and they are just answered when the educator is online. This is really time-consuming. Virtual education is good when the educator is online and immediately answers the questions*.” (P_7_).

One educator also noted:

“*If you ask me, offline virtual education cannot meet all needs. Reading just a few slides cannot help make a nurse. There is a need to establish a relationship between educators and nurses through online education.*” (P_2_).

One authority stated:

“*The majority of the courses were prepared through adding voices and sending them to senior students. Once they came in early April, they made many excuses and said that they could not open the files, and they had no access to the Internet. Unfortunately, they had not read the slides. I think students do not like to read the slides until there are some obligations. When they attend classrooms, they can have questions and answers in the presence of educators and understand the lessons better with a one-hundred-percent quality*.” (P_1_).

#### Unstable clinical education

Clinical education is an integral part of education for the nursing profession. Regarding the nursing school’s mission-centered nature and the necessity to train competent graduates to meet the organization’s needs, clinical education was maintained during the COVID-19 pandemic with the least disruption possible regarding the health protocols once the disease peak subsided. The participants mentioned cases such as training students in wards with no confirmed COVID cases, limitations in clinical education due to a reduction in the number and diversity of non-COVID-19 patients, incomplete clinical education because of the shortage of personal protective equipment, delays in clinical education for non-field students, completion of field internship programs despite the pandemic, and the reduced educational capacity of hospitals due to decline in the hospitalization of non-COVID-19 patients. The participants also mentioned a lower quality of academic courses according to the instructions by the MoHME, resistance by some students against internship, educator persuasion of students to complete internship programs, a lack of stability in clinical education programs, and a lack of fulfillment of intensive internship programs to compensate for units facing delays. A student stated:

“*In April, the conditions became better for us and the whole country, and they could give us face masks. Every five days, we could get a reusable face mask and a bottle of alcohol to use in the hospital. The given equipment was for those taking their internship courses. After one month and a half, we could continue our internship program.*” (P_6_).

One administrator noted:

“*They allocated a room for students to complete their internship programs*. *There were also boxes for clean and contaminated shoes. The students were not allowed to enter the dormitory with the shoes on in times of their internship. Two educators were further trained by the education department head to be an intermediary between us and the students and to train the students upon their arrival. The educators could also check the students once backed. The students needed to take off their contaminated shoes, wear clean slippers, and change their clothes. If they had on contaminated masks, they had to throw them into specific yellow trash bins. If the masks were N-95 ones, they had to wash them and even wash their hands and face and then enter into the dormitory.*” (P_1_).

#### Unstable theoretical education

In this study, in-person classrooms were unstable and varied with the disease’s peak. During in-person education during the disease’s reduced height, there was much emphasis on adherence to physical distancing and respect for health protocols. The participants also noted the better quality of learning during in-person education, student interest during in-person classroom sessions, interactions between educators and students as the main factor affecting better understanding, and continuous theoretical education following the prevalence of COVID-19. The participants further noted an increase in absenteeism by some students suspected with COVID-19 (two-week quarantine), resistance by some visiting educators to hold in-person classrooms, and repeated planning for theoretical education regarding new and unstable conditions. One administrator reiterated:

“*Among the challenges was that one of the visiting educators became sick. She was absent due to being infected with COVID-19. It took two weeks to replace her with someone else. This also faced the students with some problems.*” (P_8_).

One educator stated:

“*In fact, students were insisting on repeating online recordings in the classroom. They believed that they could better understand in classrooms and the PowerPoint files were not of use.*” (P_5_).

Another one noted:

“*There was no stability in the classrooms. There were cancellations, and we were required to attend classrooms some days. There was kind of uncertainty and stress, lowering the efficiency of education*.” (P_4_).

## Discussion

This study aimed to explain the impact of the COVID-19 pandemic on nursing education from administrators, educators, and students’ perspectives. The spread of this disease has thus severely disrupted life aspects, including education, while creating an unprecedented run-through in this field [20]. Naturally, the pandemic has influenced nursing education.

The first category extracted from this qualitative study was “safe management in ambiguous situation,”; which encompassed the sub-categories of “provision of health safety grounds,” “requirements for successful confrontation,” and “outcomes.” Evidence indicates that the key to deal effectively with pandemics is prevention. The experiences of affected countries also show that policies and measures intend to break the transmission chain and reduce the virus spread through lowering contacts and increasing physical distancing between healthy and suspected cases [21]. The experiences were demonstrated in one survey in which personal protection, structural protection, and safety problems were among the main themes of nurses’ experiences in self-protection domains during care for patients suffering from COVID-19 [22] that followed the two sub-categories extracted in the present study. Ulenaers et al. reported that one of the most important stressors for nursing students in clinical education was the availability and use of personal protective equipment [15] which is consistent with this study.

The second category, “perceived situation,” also consisted of “underlying conditions” and “perceived stress.” In this sense, the correct understanding of a situation is among the prerequisites toward task fulfillment and proper decision making. According to a United States survey, COVID-19 has negatively affected student life prospects. To demonstrate, 31 % of students had faced graduation delays, and 40 % of individuals had lost their jobs, internship programs, or job opportunities. Low-income students (55 %) had further encountered graduation delays more than high-income peers [23]. In one study, nursing students had also confirmed the spread of incorrect information in social networking and high-risk behaviors by people. Most feared the disease, and they were concerned about their family, so they were continually sticking to protective measures. Students had a slight level of fear regarding the risk of infection in classrooms, but they were more concerned about being affected during clinical courses. Some students also talked about problems with concentration and learning. However, all the students appreciated educators and faculty members’ support in difficult times [24]. In another study, stress levels among nursing students during the COVID-19 pandemic were reported as moderate. However, student stress levels were higher than reported in previous years. Additionally, the findings had revealed that age, gender, news feeds, concerns about the risk of infection, and restrictions on travels had affected the levels of stress in the students [13]. A survey had reported that graduates from Italian universities had experienced fear, lack of confidence, and anxiety about the future after the pandemic [25], which was in line with the present study results. A study on happiness and satisfaction levels amid the COVID-19 pandemic of 213 students enrolled in Islamic Azad University in Tehran, Iran, had demonstrated that the students had a good but moderate level of happiness and high levels of life satisfaction [26]. The survey had been conducted on groups of students in various fields, using virtual education, whose experiences were naturally different from those in the present study, receiving in-person and clinical education with wide-ranging perceptions.

The third category obtained in this study was “adaptive coping,” which takes account of the sub-categories of “experience and coping with situations” and “collaboration.” In this regard, Kim et al. reported that nursing students experienced high levels of poor mental health during the COVID-19 lockdown. High levels of resilience were associated with a lower risk of stress, anxiety, and depression, whereas high spiritual support was associated with a lower risk of depression [27]. As well, Huang et al. showed that nurses and nursing students expressed anxiety, fear, despondency, and anger during the COVID-19 pandemic. All such reactions had been higher in nurses compared with nursing students. Researchers also found a significant relationship between anxiety, fear, despondency, anger, and using coping strategies. The nurses utilized coping strategies, particularly problem-oriented ones, more than nursing students, but both groups were equal in emotion-oriented coping strategies [14]. In a phenomenological study on physicians in recovery from COVID-19 in Yazd, Iran, the positive emotional reactions included hopefulness, motivation, a sense of happiness with medicine, and regulation of emotions. The negative responses were depression, anxiety, and anger. This survey showed that the infected cases’ experiences involved adverse consequences and sometimes personal growth [28], which agreed with the present study. Ulenaers et al. reported that during the COVID-19 pandemic, more than half of the nursing students experienced an enriching, remarkable, and instructive experience during their training [15].

The fourth category, “educational facilitators and challenges,” consisted of the sub-categories of “educational facilitators” and “educational challenges.” In a similar study, all undergraduate nursing students had appreciated the educator and faculty member support during the COVID-19 pandemic [24]. The emergence of the COVID-19 pandemic led to a sudden transition to distance learning. These conditions posed many challenges for nursing students who were traditionally trained in a face-to-face learning environment. In a qualitative study conducted in the United States, themes such as technological challenges, academic relationship changes, and role stress were extracted [29]. One study reflecting on the impact of COVID-19 on India’s higher education system had further found instability in all educational activities, scientific research, professional development, and assessments. The same study also found a reduction in job opportunities and an emergence of new teaching methods [20]. In another study, most students reported access to personal computers, smartphones, and e-mails, but only 26.6 % had the advantage of high-speed Internet. The level of awareness and use of automated operating systems (here, MUELE) was also high among the respondents, but 50 % of the students lacked the skills to work with the university’s online education system. About half of the students believed that an efficient electronic education method could reduce the quality of knowledge. The internet and connectivity costs were among the most critical barriers to online learning. Participants suggested integrating online and offline strategies to moderate the challenges of high-quality access to the Internet [30]. Such challenges were similarly observed in the present study.

The fifth category in this study was “continuing education in an uncertain context,” encompassing the sub-categories of “gradual formation of education in a virtual context,” “unstable clinical education,” and “unstable theoretical education.” As with any other challenges, the COVID-19 pandemic raised many threats and opportunities for universities and scientific communities. This phenomenon helped such organizations gain valuable experiences through online education and prepare to enter a new era of education using novel methods [31]. Besides, studies revealed that using different telenursing methods such as sending messages, using video or phone calls, and mobile-based educational applications could have positive consequences for patients [32–35]. With increasing computer literacy, online learning and gaming technology for education have increased in popularity [34]. COVID-19 has thus accelerated the exploitation of digital technologies for education. Higher education institutions in India positively reacted to existing challenges during this crisis and adopted various strategies to deal with them. Benefitting from the help of the Ministry of Human Resource Development, the authorities had developed numerous virtual operating systems, electronic books, other learning-teaching products, and media and social networking (e.g., WhatsApp, YouTube, and Telegram) applications to maintain the education process [20]. Evidence had shown that imposed online education has led to some problems for older students, those living in villages, and those making a living for their families facing limited electronic resources [17]. A survey had indicated that students challenged with global lockdowns, moving towards online education, had the highest levels of satisfaction with the support provided by their university and the relations office staff [9]. Wallace et al. showed that despite the educational challenges, during the COVID-19 pandemic, skills and creativity in nursing students increased. They became self-directed. These students formed independent study groups, and practiced nursing skills. They also felt accomplishment with discovering their strengths and increasing efficiency [29]. These investigations substantiated the results of the present study.

### Limitation

One limitation of this study was that the participants were likely not to express their actual and real-life experiences. The researchers tried to establish good relationships with them, gain their trust, and emphasize their identity’s confidentiality to control this limitation.

## Conclusions

This study’s main theme was “close conflict of education with COVID-19,” which included mentioned categories. Preparing nursing students for specific competencies is needed. As a whole, researchers concluded that new complicated situations have led to instability and numerous challenges to the education process, which can be turned into opportunities through correct planning, scientific management, stabilized decision-making, unity of command, use of technologies within other educational capacities, fostering coping mechanisms, and comprehensive support for higher education institutions affected by COVID-19 to promote the education process. Finally, it crucial that nursing administrators, educators, and students communicate about matters such as educational facilitators and challenges during the COVID-19 pandemic to promote the quality of education and provide safe patient care.

## Data Availability

The datasets used and analyzed during the present study are available from the corresponding author on reasonable request.

## Abbreviations

COVID-19: Coronavirus Disease of 2019
MoHME: Ministry of Health and Medical Education
nCoV: Novel coronavirus
